# TERC haploid cell reprogramming: a novel therapeutic strategy for aplastic anemia

**DOI:** 10.1186/s10020-023-00691-w

**Published:** 2023-07-09

**Authors:** Xinyu Tang, Ruirong Xu, Yan Wang, Kaiqing Chen, Siyuan Cui

**Affiliations:** 1 Shandong University of Traditional Chinese Medicine, Jinan, 250014 China; 2 Department of Hematology, Affiliated Hospital of Shandong University of Traditional Chinese Medicine, Jinan, 250014 China; 3 Institute of Hematology, Shandong University of Traditional Chinese Medicine, Jinan, 250014 China; 4 Shandong Provincial Health Commission Key Laboratory of Hematology of Integrated Traditional Chinese and Western Medicine, Affiliated Hospital of Shandong University of Traditional Chinese Medicine, Jinan, 250014 China

**Keywords:** Telomerase RNA component, Cell reprogramming, Aplastic anemia, Telomerase

## Abstract

The telomerase RNA component (TERC) gene plays an important role in telomerase-dependent extension and maintenance of the telomeres. In the event of TERC haploinsufficiency, telomere length is often affected; this, in turn, can result in the development of progeria-related diseases such as aplastic anemia (AA) and congenital keratosis. Cell reprogramming can reverse the differentiation process and can, therefore, transform cells into pluripotent stem cells with stronger differentiation and self-renewal abilities; further, cell reprograming can also extend the telomere length of these cells, which may be crucial in the diagnosis and treatment of telomere depletion diseases such as AA. In this study, we summarized the effects of TERC haploid cell reprogramming on telomere length and the correlation between this alteration and the pathogenesis of AA; by investigating the role of cell reprogramming in AA, we aimed to identify novel diagnostic indicators and therapeutic strategies for patients with AA.

## Background

Aplastic anemia (AA) is a severe bone marrow failure disorder that is clinically characterized by pancytopenia (Gajbhiye et al. 2022). Diagnosis and treatment of AA have remained the focus of clinical attention; however, the current understanding of AA pathogenesis remains unclear. The mechanism underlying AA primarily involves immune disorders, hematopoietic stem/progenitor cell (HSPC) damage, and hematopoietic microenvironmental abnormalities. Telomere length is reduced via processes such as cell division and replication, and in recent years, this telomere loss has been closely associated with the development of AA.

Telomerase is an important enzyme in the maintenance of telomere length and prevention of telomere loss. It is predominantly composed of RNA and proteins, and is involved in apoptosis and DNA damage repair (Jaiswal et al. 2013). Among these components, the telomerase RNA component (TERC), in particular, plays an important role in maintaining telomere length. Therefore, haploinsufficiency deficiency of these gene can cause abnormal telomerase function and can affect the maintenance of telomere length; the corresponding telomere loss can affect gene transcription and cell cycle progression, ultimately leading to cell aging–related diseases, such as AA (Tsai et al. 2022a). Undifferentiated stem cells exhibit excellent multidirectional differentiation ability. Additionally, during differentiation, reduction in the telomere length of these stem cells can be moderately reduced without causing telomere dysfunction (Bloom et al. 2022). Therefore, reversing the differentiation process is beneficial for maintaining cell stemness and reducing telomere loss to a certain extent; this approach, ultimately, holds the potential to reverse the onset and progression of AA.

Cell reprogramming involves the modification of gene expression within a cell, transforming it from one cell type to another; this alteration in cell differentiation lineage can, thereby, affect the cell's ability to differentiate (Huang et al. 2022). Prior studies have established that cell reprogramming can transform TERC haploid cells into cells of other lineages; for example, mature somatic cells can be transformed into various cells, such as mesenchymal stem cells (MSCs), and induced pluripotent stem cells (iPSCs). Cell reprogramming can enhance the differentiation ability of these cells and extend telomere length; this, in turn, can alleviate or reverse telomere depletion. Therefore, cell reprogramming holds potential as a therapeutic strategy for treating various diseases (Thanaskody et al. 2022) and is, consequently, being evaluated in a growing number of studies. However, the potential role of cell reprogramming in AA has not been explored previously.Our study has the potential to stimulate further research and introduces novel ideas that could be used to establish effective diagnostic and therapeutic strategies for AA.

## Effects of TERC on telomere length

Telomeres are small DNA–protein complexes at the end of eukaryotic chromosomes (Srinivas et al. 2020); telomere repeats and telomere-binding proteins form a special “cap” structure that together maintain chromosomal integrity and control the cell division cycle (Glousker and Lingner 2022). However, this protective function is gradually lost as the cell division cycle progresses; consequently, incomplete DNA replication and inhibition of telomerase activity can occur, resulting in shortened telomeres (O’Sullivan and Karlseder 2010), which cause telomere-related diseases such as AA.

Telomerase is an important component of many factors that can affect telomere length (Telomeres 2018). As a nucleoprotein reverse transcriptase, telomerase is primarily composed of TERCs and proteins that can replenish the telomeres lost during DNA replication, repair and lengthen telomeres, and reduce telomere loss during cell division. Overall, telomerases play important roles in maintaining telomere stability, genomic integrity, and long-term cell viability (Simonsen et al. 2002). Moreover, telomerase is also involved in tumor progression and cancer cell survival. Human telomerase reverse transcriptase (hTERT) expression promotes the aggressiveness of cancer cells (Jaiswal et al. 2017) and can influence tumor progression by upregulating the expression of TSPAN13 (Jaiswal et al. 2018); alternatively, hTERT knockdown can reduce urokinase-type plasminogen activator (uPA) expression and reverse the epithelial–interstitial transition of cancer cells (Jaiswal and Yadava 2019). In addition to maintaining and lengthening telomeres, telomerase can alter the post-translational level of proteins; furthermore, it can influence stem cell function, enhance epithelial cell activity, regulate genome stability, regulate telomerase-related genes to modulate apoptosis and proliferation levels, and regulate telomerase-related molecules to target specific pathogenic processes (Jaiswal and Yadava 2019). Therefore, it is important to fully understand the regulatory mechanisms underlying telomerase activity.

Telomerase is not widely distributed throughout the body; its activity is strongly regulated in normal human tissues, and active telomerase is only detected in cells with strong proliferative abilities, such as hematopoietic stem cells (HSCs) and germ cells. In most other cell types, telomerase activity is inhibited and gradually terminated as the cells undergo differentiation and maturation. When telomerase-positive cells transition from an activated to a quiescent state, they exit the cell cycle and downregulate telomerase expression through transcriptional inhibition pathways, thereby affecting telomerase proteolysis. Under physiological conditions, reduced telomerase activity in cells leads to continuous cell division and telomere shortening, which eventually results in cellular senescence and death (Holt et al. 1999). Notably, telomerase is highly active in most human tumors; when these tumors develop, telomerase can be reactivated and can, consequently, participate in the malignant transformation of the tumor cells (Holt et al. 1999). In the field of clinical research, it is crucial to ascertain the mechanisms behind how this expression pattern is maintained in adult cells for an extended period and to determine how this dysregulation of expression can be reversed during tumor progression.

**Fig. 1:**
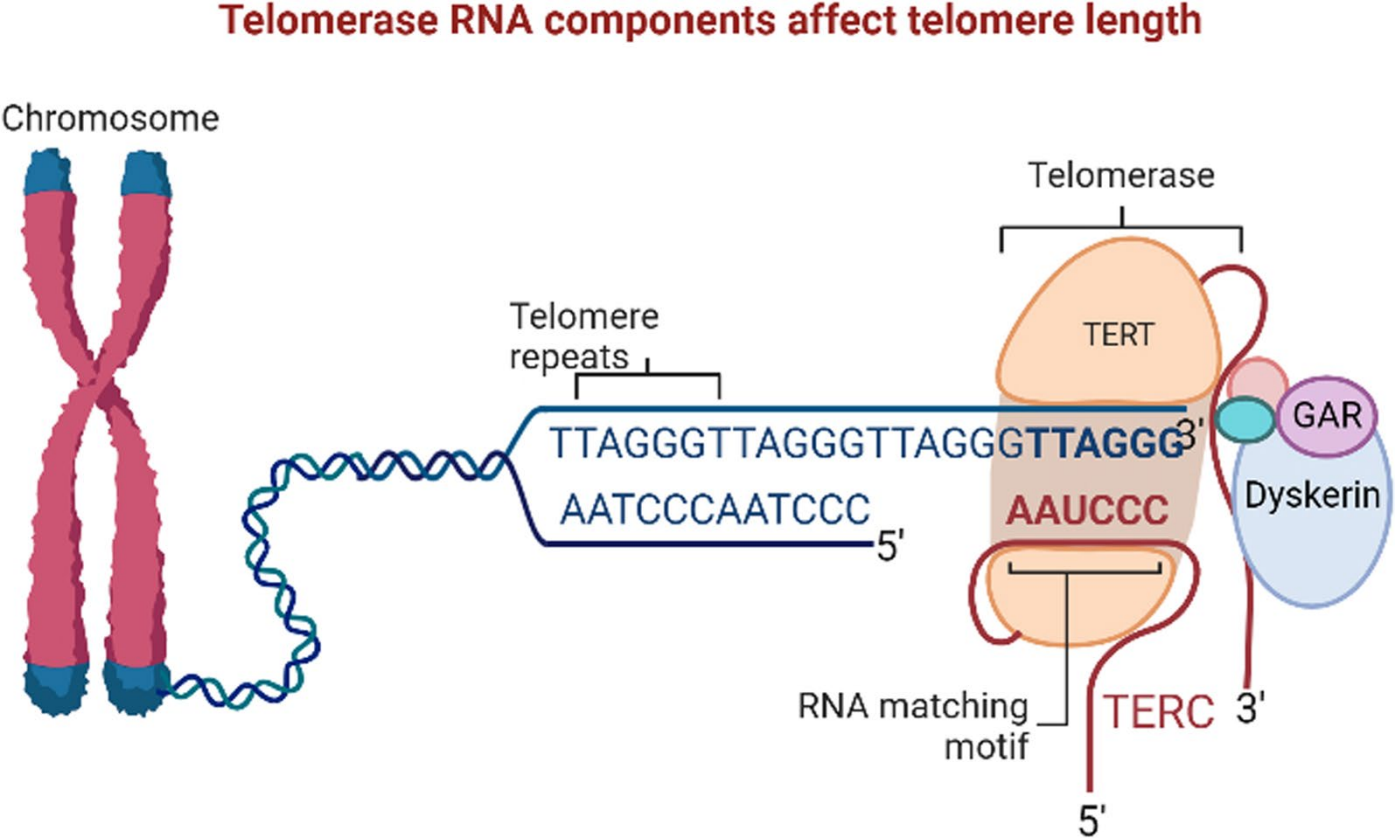
Telomerase RNA component (TERC) influences telomere length by affecting telomerase activity. Telomerase plays an important role in maintaining the stability of telomere length. Telomerase is primarily composed of RNA and protein components; among which, TERC provides a template for telomere DNA synthesis, maintains the catalytic activity of telomerase, and affects telomere length

TERC is the RNA fraction of telomerase, and is particularly important for maintaining telomerase catalytic activity, cell differentiation capacity, and chromosomal stability (Fig. 1). Specifically, it provides a template for telomere DNA synthesis, which is used by TERT to synthesize repetitive sequences of telomeric DNA; this telomere replenishment helps to maintain genome stability and long-term cell function (Shay and Wright 2019), and holds potential for the diagnosis and treatment of telomere diseases, such as AA.

However, human TERCs lack sequence conservation; additionally, it is difficult to resolve telomere loss by relying solely on the supply of telomerase. Thus, improving this telomerase deficiency has become an important goal of current research.

The core promoter of TERC/TERT consists of 260 bp with multiple transcription factor-binding sites that interact with relevant regulators (Killela et al. 2013; Kyo et al. 2008). A prior clinical study (NCT00071045) elucidated the relationship between mutations in the TERC and TERT promoter regions and the pathogenesis of AA; further, they determined that AA patients were exposed to a heterozygous mutation in the CCAAT box (CCAAT > GCAAT) located at position − 58 to − 54 in the TERC core promoter region (Aalbers et al. 2012). Reversing these promoter region mutations may, therefore, aid in the clinical treatment of AA.

Telomeres consist of hundreds of thousands of TTAGGG repeats; mutations in the telomerase complex can result in limited telomere maintenance and telomere loss (Calado and Young 2009a). Short telomeres activate programmed apoptotic mechanisms, preventing cell damage and carcinogenesis; these short telomeres may be associated with various tumor-associated sequences mutations in telomere-associated genes (Mirabello et al. 2010). Genes that regulate telomeres and telomerases play a vital role in limiting the proliferation of tumor cells while extending the lifespan of normal cells. The clinical diagnostic strategy for telomeropathy is based on screening for mutations with in the telomerase promoter region. Recent genome-wide association studies have determined that single nucleotide polymorphisms (SNPs) in genes encoding telomere-associated proteins (RTEL1 and TERT-CLPTM1) are strongly associated with cancer risk; further, in patients with tumors, telomere length is significantly associated with SNPs in the telomere-associated genes MEN1, MRE11A, RECQL5, and TNKS (Mirabello et al. 2010). PS3/BS3 is an important indicator for the detection of telomere biology disorder in individuals with TERT/TERC mutations (Nelson et al. 2023). Alternatively, some strategies for detecting telomere length and telomere-related protein expression have been developed for patients with AA.

## Effect of TERC haploinsufficiency on hematopoietic function in AA

Telomere shortening is an important inhibitory mechanism for continuous cell division. However, very short telomeres may contribute to telomere fusion and genomic instability (Costoya and Arce 2022); further, telomerase mutations are considered genetic risk factors for acquired AA (Yamaguchi et al. 2005). This Genetic dysfunction can impair telomere maintenance, leading to excessive telomere shortening and bone marrow (BM) failure, the most common and severe feature observed in patients with AA (Calado and Young 2009b). Various studies have addressed the relationship between AA and telomeres. Wang et al. (2014) confirmed the presence of shortened telomeres within the cells of patients with severe aplastic anemia (SAA); these shortened telomeres indicated an increased hematopoietic stress response in the BM, which, in turn, leads to HSC deficiency, low BM hematopoietic function, and AA development.

Wang et al. (2018) determined the expression levels of CD28, CD158, and CD70 to evaluate the function of T lymphocytes compared to the relative telomere length (RTL) of different subtypes of T lymphocytes in SAA patients and healthy subjects. They established that T lymphocyte marker expression was abnormally elevated in SAA patients compared with that in healthy subjects. Further, the RTL of T lymphocytes was significantly shortened in patients with SAA, indicating that telomere shortening was associated with T cell hyperfunction, thereby revealing the possible pathogenesis of SAA.

**Fig. 2:**
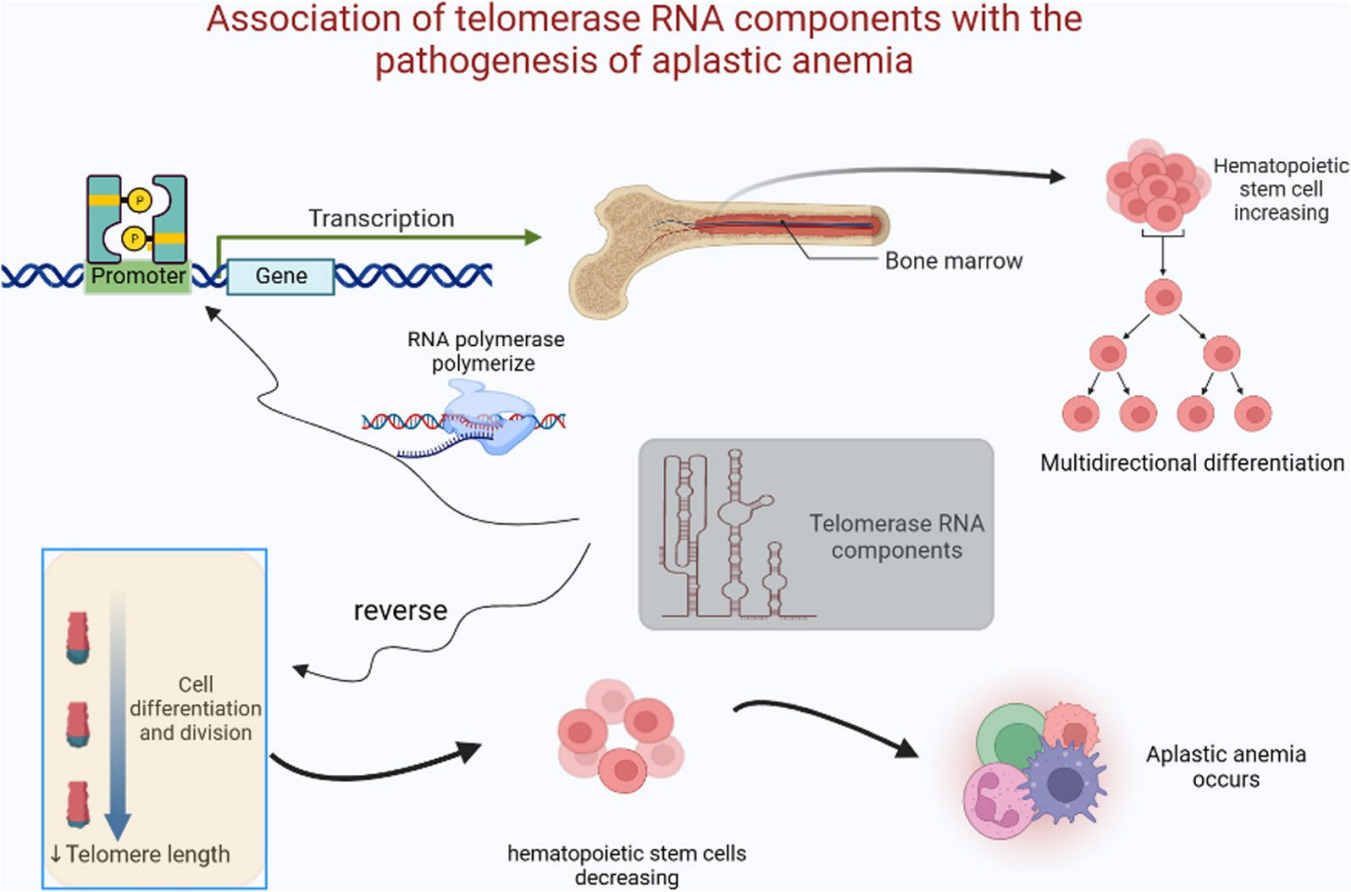
Relationship between telomerase RNA component (TERC) and pathogenesis of aplastic anemia (AA). The process of cell division differentiation is often accompanied by telomere shortening; this leads to a decrease in hematopoietic stem cells in the bone marrow, which in turn leads to AA. TERC can recruit RNA polymerase to the promoter region of bone marrow genes and regulate myeloid gene expression by recruiting transcription mechanisms; this stimulates a powerful myeloid-promoting response, resulting in an increase in the number of hematopoietic stem cells produced, which could be used to improve the hypohematopoietic function of cells in AA patients

TERC can recruit RNA polymerase to the promoter region of BM genes and regulate myeloid gene expression by recruiting transcriptional mechanisms. Ultimately, this process induces a powerful myeloid-promoting response (García-Castillo et al. 2021), which is expected to be an effective strategy for improving the hypohematopoietic function of cells in patients with AA (Fig. 2). The TERC-dependent promotion of HSCs can increase telomerase activity, increase the multidirectional differentiation and self-renewal of HSCs, and maintain HSCs homeostasis. Furthermore, TERC activity increases with an increased demand for new blood cells, which is conducive to initiating the production of new progenitor cells, with re-differentiation towards desired cell lines (Udroiu and Sgura 2021). However, this mechanism possesses certain limitations: sustaining the functional activity of TERC proves difficult, and when further multidirectional differentiation occurs, telomerase levels in the body are difficult to maintain.

Haploinsufficiency refers to the mutation of one of two chromatids in one gene of a diploid organism; under such conditions, the corresponding gene on the other chromatid is considered normal, but the expression levels of this gene product are insufficient to maintain effective functional levels, thereby affecting normal transcription and other processes. Phenotypic abnormalities in telomere biology can be detected in patients with AA who harbor TERC or TERT mutations (Yamaguchi et al. 2003). Carvalho et al. (2022) determined that genes involved in telomere biology, such as TERC, are mutated in patients with AA, leading to haploidinsufficiency. This haploinsufficiency may then cause cell cycle arrest, HSC loss, and rapid BM failure, resulting in dysfunctional cell regenerative ability, which in turn leads to cellular aging diseases such as AA (Logeswaran et al. 2021). TERC haploinsufficiency can also cause telomere shortening; this phenomenon affects the proliferative capacity and genomic stability of HSCs, thereby depleting the stem cell pool (Thongon et al. 2021), which can result in BM failure and severe AA. Improving telomerase function and maintaining telomere length are extremely important in the treatment of AA. At present, the methods for prolonging telomere length mainly include cell reprogramming, modulation of the cell cycle, regulation of cell proliferation and apoptosis, control of telomere-related genes, and manipulation of protein interactions at the post-translational level (Li 2011). In this review, we primarily explored the mechanism and corresponding application of cell reprogramming, which can be used to alleviate AA by the lengthening of telomeres; thus, we not only highlight novel therapeutic strategies for AA but also lay a foundation for the treatment of other telomere-related diseases.

## Cell reprogramming alleviates AA hematopoietic failure

Cell reprogramming is an important therapeutic strategy that influences normal cellular function. It can affect cell development, alter gene expression, trigger transformation into different cell type, and enhance stem cell activity, enabling them to evade immune responses and respond to injury-mediated stress; this, ultimately, provides the possibility of cellular regeneration via cell reprogramming. When telomere-related gene mutations and haploinsufficiency occur, the insufficient gene product expression can be altered by the reprogramming of differentiated mature cells into cells with higher differentiation potential; consequently, TERC activity can be improved and telomere length can be extended, which could potentially provide clinically therapeutic benefits to patients with AA (Argaez-Sosa et al. 2022). Cell reprogramming can play a therapeutic role by altering cell fate via transcription factor regulation and forced expression of non-coding RNA, which transforms somatic cells from one lineage to another (Ma et al. 2021), thereby providing them with a stronger ability to proliferate and differentiate than primary cells.

Depending on whether this process requires an intermediate pluripotency transition, cell reprogramming can be divided into direct and indirect reprogramming (Tang et al. 2017). Direct reprogramming, also known as transdifferentiation, does not require the transition to intermediate pluripotent states and is, therefore, more efficient (Gascón et al. 2017). Indirect reprogramming requires the transition to an intermediate pluripotent state before lineage conversion. Due to the strong self-renewal ability of pluripotent stem cells, indirect reprogramming can produce target cells on a large scale, which is more suitable for ex vivo cell production (Wang et al. 2023). Direct reprogramming can occur in situ in the target tissues and is, therefore, more suitable for tissue repair in vivo. In addition, unlike indirect programming, direct reprogramming can preserve the epigenetic features of the origin cells within the reprogrammed cells, including as senescence features, which enables these reprogrammed cells to mimic those found in aging-related diseases (Gascón et al. 2017).

**Fig. 3:**
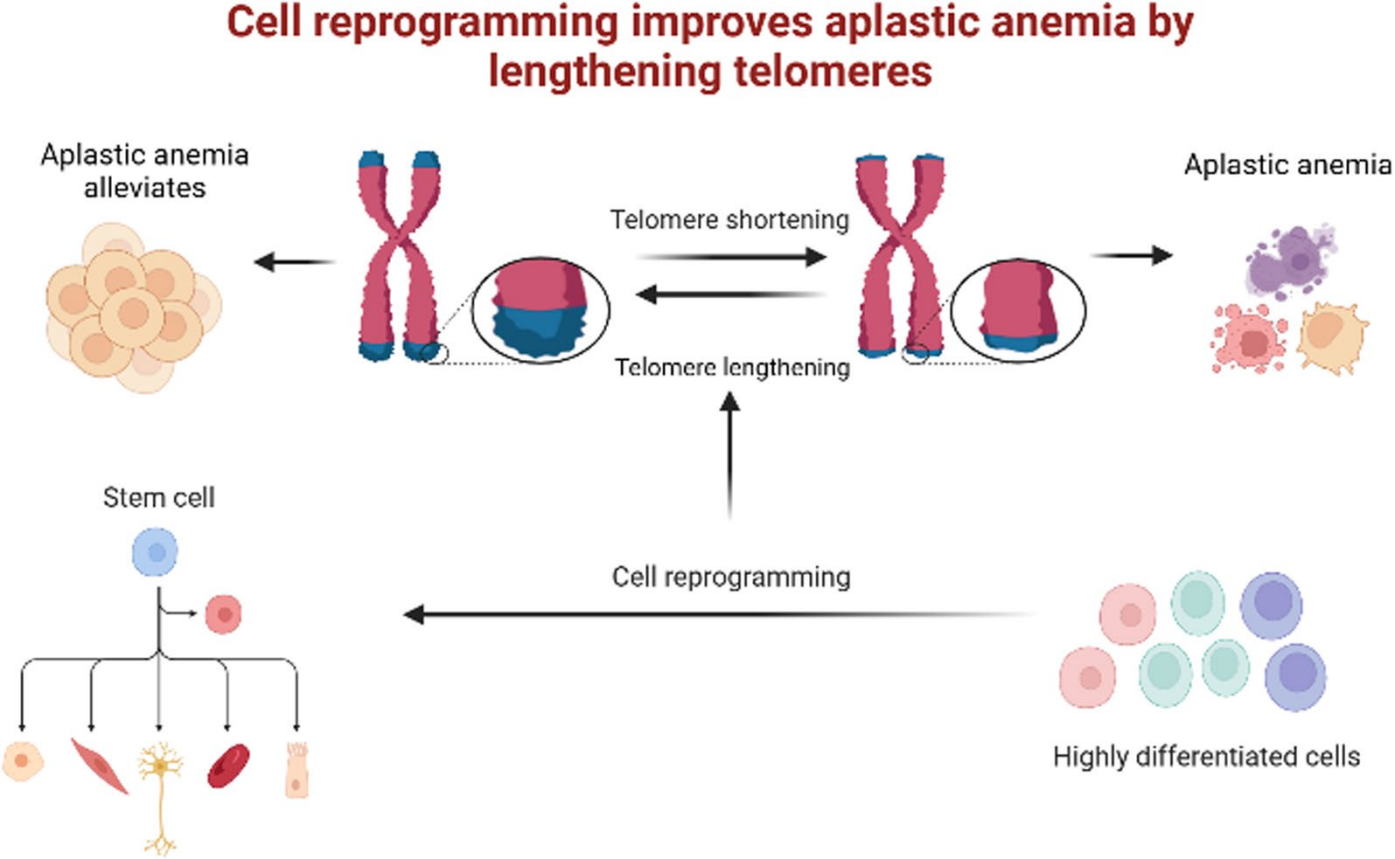
Cell reprogramming treat aplastic anemia (AA) by increasing telomere length. Cell reprogramming is an important way of transforming the cell types of differentiated cells; this process can generate pluripotent stem cells and achieve multidirectional differentiation. Telomere depletion is one mechanism that results in AA development. Through cell reprogramming, cells with stronger differentiation ability can be obtained, with extended telomeres and reduced telomere loss; this has the potential to confer a therapeutic effect on AA

HSCs are responsible for producing various cell types via self-renewal, replication, and multidirectional differentiation; however, AA-associated BM failure is predominantly caused by defects in these HSCs (Wang et al. 2023). Upon disruption of HSC function, maintaining a sufficient quantity of blood cells to fulfil the body's requirement becomes difficult; this may, ultimately, result in the development of AA (Li et al. 2020). Clinically, HSC transplantation is mainly used to compensate for the lack of HSCs in patients with AA. However, there are complications associated with transplantation, such as graft-versus-host disease and risk of infection; therefore, the prognosis for patients with AA remains unsatisfactory (Liu et al. 2022). The identification of cells with strong differentiation abilities would be extremely beneficial in the development of novel therapeutic strategies in clinical practice. Overall, cell reprogramming, which increases telomere length and improves BM hematopoiesis, may be an important mechanism for reversing hematopoietic failure in patients with AA (Fig. 3).

To achieve in vivo and in vitro reprogramming, it is important to identify appropriate origin cells. Currently, studies assessing the efficacy of cell reprogramming in the treatment of various diseases are being conducted. For example, cell reprogramming has been determined to reverse myocardial damage, improve cardiac function, alleviate eye diseases, and reprogram astrocytes to promote neuronal repair of the central nervous system (Ye et al. 2018; Mandai et al. 2017; Sundberg et al. 2013). The plasticity observed in adult somatic cells, alongside their ability to differentiate into specific lineages following reprogramming, holds promise for replacing damaged or abnormal tissues (Bazina et al. 2021); ultimately, this may provide new opportunities for the clinical treatment of AA.

## Clinical strategies for improving TERC function via cell reprogramming in the treatment of AA

Telomere haploinsufficiency affects telomere-related gene expression levels, impairs genomic stability, leads to cell cycle arrest and BM failure, reduces TERC activity, accelerates telomere shortening, reduces HSC count, and triggers AA progression (Logeswaran et al. 2021). This is an important mechanism that hinders AA therapy; nonetheless, emerging cell reprogramming strategies are expected to be helpful in overcoming this complication. Reprogramming differentiated cells into stem cells with higher differentiation potential allows these cells to re-differentiate, self-renew, and replicate; ultimately, this improves the functional activity of TERCs, extends telomere length, and produces more effective HSCs, which can alleviate the BM hematopoietic failure of AA.

### Treatment of AA via MSC cell reprogramming

Stem cells changes are key targets of tumor transformation; correspondingly, there is an inextricable link between normal stem cells and cancer stem cells (Dick 2003). The self-renewal and differentiation abilities of stem cells provides them the potential for use in clinical treatment, which has been effectively demonstrated in MSCs. MSCs possess superior differentiation abilities than HSCs and can differentiate into a variety of cell types, including those in the bone, fat, and muscle (Ding et al. 2022). However, MSC telomerase lacks immortalizing activity and cannot maintain telomere stability for prolonged periods. Therefore, the differentiation and renewal ability of MSCs is gradually lost during cell passage, resulting in replicative aging and rendering them susceptible to tumor transformation (Zimmermann et al. 2003). This is a key complication affecting the development of novel anti-cancer therapies and the use of MSCs in stem cells transplantation programs; moreover, upon the occurrence of haploinsufficiency, the effectiveness of this therapeutic strategy decreases further.

MSCs are a key component of the BM microenvironment; therefore, MSC dysfunction is associated with impairment of the BM microenvironment (Xia et al. 2020). HSCs in patients with AA are prone to differentiate into adipocytes, resulting in a decrease in the niche of HSCs. This may cause an abnormal hematopoietic microenvironment, affect the proliferation and differentiation of HSCs, and participate in the pathogenesis of AA. Telomere haploinsufficiency can also affect cell cycle progression and interfere with cell proliferation and apoptosis; this may, ultimately, lead to impaired TERT and TERC functions, reduced HSC counts, and BM failure. MSCs can effectively regulate hematopoiesis, particularly in preventing the replacement of BM hematopoietic cells by adipocytes in patients with AA. MSCs also improve the hematopoietic microenvironment, and promote BM hematopoiesis; therefore, maintenance of a normal number and optimal activity of MSCs is essential for the recovery of hematopoietic function (Srivastava et al. 2022).

MSCs possess wide therapeutic potential and are often used in combination with HSC transplantation in the treatment of AA. The corresponding results demonstrate that the transfusion interval of AA patients after 3 months of combined MSC/HSC treatment was significantly longer than that before treatment. Further, the peripheral blood leukocyte, lymphocyte, and platelet counts levels were significantly elevated; the CD4/CD8 ratio was improved; the risk of graft-versus-host disease was relatively low; and the 5-year survival rate of the patients was high. Ultimately, this indicated that combined MSC/HSC treatment is an effective and safe treatment option for patients with AA (Sheng et al. 2022).

However, the MSCs currently used for clinical treatment are primarily derived from adult or fetal tissues; these MSCs have some limitations including low purity, insufficient sources, high cell senescence, and reduced multidirectional differentiation and proliferation capacity (Fujii et al. 2018). In addition, tissue-derived MSCs lack TERC expression, and the regulation of telomerase genes is affected by a variety of factors (Gonzaga et al. 2022); consequently, the function of these MSCs is restricted. Telomere haploinsufficiency is a key problem; its occurrence leads to a lack of telomere-related gene products, weakened TERC activity, insufficient cell regenerative ability, and the failure of MSCs libraries. To overcome these limitations, alternative methods must be employed to obtain the large number of high quality MSCs needed (Kimbrel et al. 2014). To solve these problems, researchers have turned their attention to cell reprogramming to generate large quantities of high-quality MSCs (Jiang et al. 2019). Embryonic stem cells (ESCs) are effective sources of reprogrammed MSCs (Davies et al. 2017). Compared with MSCs from other tissue sources, MSCs obtained via cell reprogramming exhibit advantages in terms of proliferative differentiation and modulation of immune activity (Monsarrat et al. 2016). Cell reprogramming is an effective way of mitigating the loss of MSCs and producing more functional MSCs (Vermeulen et al. 2022). Therefore, this strategy is expected to become the focus of research in the field of regenerative medicine.

Milena et al. postulated that MSCs in the BM play a pivotal role in cell reprogramming. They suggested that MSCs can induce transcriptional upregulation and nuclear translocation of activated transcription factor 3 (ATF3) in HSCs; this process promotes the formation and continuous multidirectional differentiation of myeloid progenitor cells, thereby improving hematopoietic function (Perrone et al. 2022). MSC transplantation not only restores the function of host MSCs but also reprograms host macrophages towards the arginase 1-positive phenotype with tissue repair functions, restores the BM microenvironment, and inhibits leukemia development (Xia et al. 2020). Therefore, the use of MSCs as a model for telomerase gene regulation and cell reprogramming in normal human adult stem cells could potentially provide promising therapeutic strategies; however, research on the effect of MSC cell reprogramming for the treatment of AA is limited.

### Treatment of AA via iPSC cell reprogramming

In the human body, ESCs exhibit higher telomerase expression levels than those in other cells; this elevated telomerase expression helps to maintain telomere length, establish pluripotent phenotypes, and achieve multidirectional differentiation (Thomson et al. 1998). Moreover, fibroblasts and T lymphocytes can fuse with ESCs and express genes related to pluripotency, rendering the reversal of cell differentiation through somatic cell reprogramming possible. However, the use of ESCs is associated with several limitations. First, treatment with ESCs predisposes recipients to immune rejection, which necessitates the life-long administration of immunosuppressants to maintain efficacy; further, the use of ESCs in clinical research raises important ethical issues. Thus, iPSCs are important candidates for regenerative therapy (Abbar et al. 2020).

iPSCs can be produced via somatic cell reprogramming and do not affect the host immune system after transplantation. However, the reprogramming efficiency of iPSCs is affected by many factors, such as culture time, expression of homogeneous molecules, and the expression levels of various genes, such as NANOG; these factors may interfere with the reprogramming process and limit therapeutic efficacy (Onfray et al. 2022). Since the introduction of this technology, several observational studies have explored its effects on heart, ophthalmic, and neurological diseases (Ye et al. 2018; Mandai et al. 2017; Sundberg et al. 2013), confirming the therapeutic efficacy of iPSCs produced via cell reprogramming.

Telomere haploinsufficiency is an important factor affecting the activity of TERC and loss of telomere length. Winkler et al. obtained iPSCs from AA patients or patients carrying heterozygous mutations in the TERC telomerase gene. In this study, iPSCs were able to upregulate the expression of TERCs and extend telomere length; however, this accelerated rate of telomere lengthening is reduced when a TERC mutation is introduced to the iPSCs. Further, telomerase-mutated iPSCs exhibit defects in hematopoietic differentiation and may cause human telomere disease (Winkler et al. 2013). This suggests that iPSCs are essential for maintaining telomerase activity and protecting hematopoietic differentiation and that reversing telomerase mutations and TERC defects may assist in the treatment of telomerase-associated diseases. Similarly, Takahashi et al. established that telomerase expression is upregulated during iPSC production (Takahashi et al. 2007) and that its expression is closely related to telomere elongation in iPSCs (Marion et al. 2009). Tsai et al. used fibroblasts with TERC from the tail tip of mice as donor cells for somatic cell nuclear transplantation; the telomeres of these derived ESCs were determined to be significantly elongated (Yehezkel et al. 2011).

Overall, cell reprogramming is a favorable strategy to address haploinsufficiency; the use of cell reprogramming techniques to change telomere length and differentiation capacity of cells is an invaluable tool for studying telomere dynamics and reversing disease pathogenesis. Notably, although a singular round of single-cell metabolically labeled new RNA tag sequencing has been observed to reprogram TERC haploid cells into ESCs states and lengthen the telomere length of these cells, repeated steps of this process do not yield additional benefits (Yehezkel et al. 2011). Therefore, the feasibility of using this method in clinical treatment requires further validation.

Researchers are actively exploring the use of iPSCs in the study of telomere dynamics. For example, Li-Kuang et al. replicated telomere dynamic abnormalities observed in telomere-related diseases; they then successfully manipulated cell differentiation in response to these abnormalities, resulting in extension of telomere length. This, ultimately, provided a way by which the factors related to tissue dysfunction can be further evaluated and holds promise for improving telomere depletion in patients with AA (Tsai et al. 2022b). It is important to note that the use of iPSCs to treat diseases first requires isolating somatic cells and reprogramming them into a pluripotent state, followed by differentiation into different cell lineages. Nonetheless, iPSC-derived autologous cell therapy has yielded promising clinical results (Blau and Daley 2019).

As direct reprogramming can be used to generate reprogrammed cells in situ in diseased organs in animal models, its use has the potential to overcome technical difficulties associated with iPSCs, such as in vitro reprogramming and mass expansion (Srivastava and DeWitt 2016). However, human cells often have stable epigenetic properties and it induction of human pluripotent stem cells by reprogramming is considered relatively challenging (Guan et al. 2022). Altering the plasticity and regenerative ability of cells via reprogramming serves as a fundamental basis for the generation and application of iPSCs in human biomedicine. This advancement provides a strong foundation for the development of regenerative therapeutics, offering the potential to transform the fate of human cells; furthermore, it offers a promising strategy for the diagnosis and treatment of AA.

## Conclusions

Telomerase activity disorders in humans are a key cause of a various telomere-related diseases, including AA (Calado and Young 2009b). The pathogenesis of AA is complex and has proven difficult to elucidate; nonetheless, changes in telomere length and telomerase functional activity are possible mechanisms that lead to the onset of AA. Telomerase is primarily composed of RNA and proteins, among which, the RNA component of telomerase, TERC, can maintain telomere length. Great differences in TERC activity have been observed in normal cells and diseased cells and this phenomenon has been associated with the occurrence of AA. Through several studies, it has been established that haploinsufficiency of the telomerase TERC gene affects the functional activity of telomerase, impairs its ability to maintain telomere length, and leads to further shortening of telomeres over the course of the cell cycle. Such alterations can result in the loss of protection of chromosomal ends, leading to DNA damage, activation of DNA damage signaling pathways, and formation of non-homologous chromosomal end junctions (Winkler et al. 2013), which can ultimately contribute to the development of various diseases. The potential of correcting TERC haploinsufficiency to alleviate telomere depletion highlights a novel pathway for treating various diseases.

Cell reprogramming is an important method for altering cell type by adjusting the corresponding cell differentiation, and is primarily implemented via direct or indirect reprogramming, cell reprogramming can address the issue of TERC haploinsufficiency, extend telomere length, and improve the ability of TERC in the maintenance of telomerase activity. Based on this understanding, cell reprogramming may reverse telomere loss in patients with AA by increasing telomere length, maintaining the integrity of epigenetic transcriptional regulation, and limiting telomere shortening during replication. Nonetheless, establishing an appropriate reprogramming pathway according to the characteristics of different cells and the disease itself will be necessary in future studies.

Cells with strong multidirectional differentiation can be used as target cells for cell reprogramming, including MSCs and iPSCs. These cell types enable multidirectional differentiation, participate in various cellular processes, protect telomerase activity, maintain telomere length, and reduce the incidence of telomere depletion–related diseases. Introducing transcription factors into the somatic cells of patients and reprogramming them into pluripotent stem cells can confer similar differentiation abilities to ESCs whilst avoiding the ethical issues of ESCs. Therefore, this strategy holds great potential in disease simulation, drug screening, and cell therapy, representing a promising advancement in the field of cell therapy. Considering the benefits of cell reprogramming in other systemic diseases, its application in AA is also worth investigating as it holds the potential to reduce AA caused by telomere loss and could provide a new direction for clinical treatment. Although there is limited research into the application of cell reprogramming for the treatment of AA, this review offers novel ideas that have the potential to shape the future development of AA-targeted therapeutics.

## Data Availability

Not applicable.
